# The Effect of *Salvia hispanica* and *Nigella sativa* Seed on the Volatile Profile and Sensory Parameters Related to Volatile Compounds of Dry Fermented Sausage

**DOI:** 10.3390/molecules27030652

**Published:** 2022-01-19

**Authors:** Paula Borrajo, Małgorzata Karwowska, Jose M. Lorenzo

**Affiliations:** 1 Centro Tecnológico de la Carne de Galicia, Rúa Galicia No. 4, Parque Tecnológico de Galicia, San Cibrao, das Viñas, 32900 Ourense, Spain; paulaborrajo@ceteca.net (P.B.); jmlorenzo@ceteca.net (J.M.L.); 2 Department of Animal Food Technology, Sub-Department of Meat Technology and Food Quality, University of Life Sciences in Lublin, Skromna 8, 20-704 Lublin, Poland; 3 Facultad de Ciencias de Ourense, Área de Tecnología de los Alimentos, Universidade de Vigo, 32004 Ourense, Spain

**Keywords:** fermented sausage, *Salvia hispanica* seed, *Nigella sativa* seed, volatile compounds, flavor, odor

## Abstract

The aim of the study was to evaluate the effects of *Salvia hispanica* and *Nigella sativa* seed addition on the volatile compounds and sensory characteristics (with particular emphasis on odor and flavor) of traditionally produced dry fermented sausages with reduced nitrites. Five different sausage formulations were prepared: control sample; samples with 1% and 2% addition of chia seed; samples with 1% and 2% addition of black cumin seed. The sausages were subjected to analysis including proximate chemical composition, volatile compound determination, and sensory analysis. The sausages with chia seed in the amounts of 1% and 2% as well as the sample with 1% addition of black cumin seed were characterized by positive sensory features, and their overall quality was rated above 7 c.u. on a 10-point scale, similar to the control sausage. Sausage samples with the addition of cumin seed were characterized by the highest herbal odor and flavor. The addition of *Salvia hispanica* and *Nigella sativa* seed significantly affected the amount of volatile compounds in fermented sausages. Sausages with black cumin presented the greatest amount of total volatile compounds, mainly contributed by terpenes.

## 1. Introduction

Among the many types of meat products, dry-fermented sausages have a special significance for consumers as these meat products are considered healthy and safe foods [1]. An additional value for increasing their nutritional value may be reducing or eliminating synthetic additives, mainly nitrites, as well as the addition of plant additives which have antioxidant and health-promoting properties. These activities are part of strategies to improve the nutritional value of meat products [2]. The potential pro-health effects can be attractive for consumers as the demand for healthier foods has been growing in recent years [3].

*Salvia hispanica* (chia) and *Nigella sativa* (black cumin) seeds are known as natural sources of bioactive compounds [4,5,6,7]. The biological activity of *Nigella sativa* seed has been associated with tocopherols, vitamin A and C, β-carotene, thymoquinone, saponins, and alcaloids [4]. These bioactive compounds determine the therapeutic effects of black cumin, including anti-inflammatory, anti-allergic, anti-cancer, hypoglycemic, antioxidant, hypotensive, and hypolipidemic properties. The research of Bordoni et al. [5] showed anti-Inflammatory properties of *Nigella sativa* oil related to the thymoquinone content, but also on other antioxidant components of the oil. As related by Zielińska et al. [6], black cumin plays a supporting role in the treatment of autoimmune diseases, including rheumatoid arthritis. The food industry is also paying attention to the use of the *Salvia hispanica seeds*, which are a natural source of omega-3 and omega-6, fiber, proteins, and natural antioxidants such as polyphenols [8]. The study performed by Ghaafoor et al. [9] showed that myrcetin, and rosmarinic, 3,4-dihydroxybenzoic, caffeic, and gallic acids are the key phenolic components in chia seeds. As *Salvia hispanica* and *Nigella sativa* seeds offer human benefits beyond their nutritional value, they are suitable candidates for use in meat products. Our previous study on the effects of chia and black cumin seed in traditionally produced dry fermented sausages with reduced nitrites on the changes in physicochemical parameters related to food safety [10] indicated that this plant material can be used as a natural additive in the production of fermented sausages. However, there is a lack of studies assessing the effect of the mentioned seeds on the volatile organic compounds (VOCs) in the fermented meat products. The formation of volatiles in fermented meat products is the result of complex chemical and biochemical changes in the components of meat and added non-meat ingredients, including reactions between lipids, proteins, and carbohydrates induced by bacterial or endogenous enzymes [11,12]. Lipids, naturally present or added into foods, play an important role in flavor and aroma development of meat products. They are responsible for many desirable characteristics of foods [13,14]. It is well-known that lipid oxidation causes deterioration of fatty tissues in meats, leading to a loss of quality due to the reduction of omega-3 and omega-6 fatty acids [15]. Moreover, off-flavors can develop due to their susceptibility to oxidative reactions [16]. Consequently, numerous volatile compounds are generated, such as alcohols, aldehydes, and ketones. During the ripening, the lactic acid bacteria (LAB) are involved in the formation of alcohols and aldehydes [17]. They are also responsible for the organoleptic properties such as flavor, taste, color, and texture in the dry-fermented sausages. Lipolysis and proteolysis are the main biochemical changes that occur during the ripening process. They are also implicated in the synthesis of compounds that affect the aroma, flavor, and taste [10]. Additionally, ingredients added during production can alter the volatile compounds in fermented sausage [18].

We hypothesized that the addition of *Salvia hispanica* and *Nigella sativa* seed during the production of fermented sausages with reduced nitrite will have a positive effect on the volatile organic compounds, and thus on the sensory characteristics. Thus, the objective of the study was to evaluate the effects of *Salvia hispanica* and *Nigella sativa* seed addition on the volatile compounds and sensory characteristics of traditionally produced dry fermented sausages with reduced nitrites. To our knowledge, there is a lack of studies assessing the effect of the mentioned seeds on the volatile organic compounds in the fermented meat products. The obtained results will give us the opportunity to evaluate the possibility of the practical use of these seeds in the production of meat products with reduced nitrite content.

## 2. Results

### 2.1. Proximate Chemical Composition of Dry Fermented Sausages

The proximate chemical composition of fermented sausages is presented in Table 1. As expected, all samples showed a similar salt and collagen content. Statistically significant differences between the samples were noted for the fat, protein, and moisture content (*p* < 0.05). The fat concentrations of sausage samples with added seeds were significantly higher compared to control sample (SK). The addition of black cumin seeds resulted in a significantly higher water content in the finished product, while the sausage samples with chia seeds had the same moisture content compared to the control sample. The samples with the highest amount of black cumin seeds (SBC 2%) had the highest water content and at the same time the lowest protein content among all the experimental sausages. The control sausage sample, as well as samples with chia seed addition, were characterized by the highest protein content in the range of 51.01–52.52%.

### 2.2. Volatile Compounds in Dry Fermented Sausages

**Figure 1:**
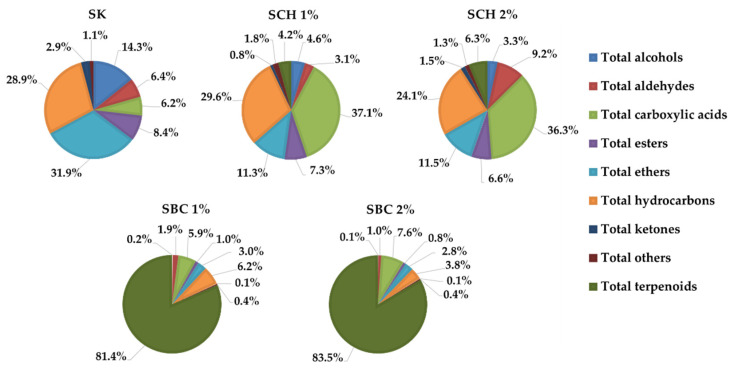
Volatile families of the dry-fermented sausages. SK—control sample; SCh 1%—sample with 1% addition of chia seed; SCh 2%—sample with 2% addition of chia seed; SBC 1%—sample with 1% addition of black cumin seed; SBC 2%—sample with 2% addition of black cumin seed.

A total of 108 volatile compounds were identified in fermented sausages after 30 days of ripening. They were classified according to their functional group: 12 alcohols, 12 aldehydes, 4 carboxylic acids, 7 esters, 7 ethers, 34 hydrocarbons, 7 ketones, 18 terpenoids, and 7 compounds classified as ‘other’ (Table 2). In general, practically all the detected volatile compounds showed significant differences (*p* < 0.05) among treatments. SBC sausages presented the greatest amount of total volatile substances, mainly contributed by terpenes, which achieved values of total compounds above 200 AU × 10^6^ g^−1^. However, the SK and SCh batches displayed values between 41 and 47 AU × 10^6^ g^−1^. On the other hand, terpenes represented more than 81% of the total compounds in SBC samples. However, in SCh sausages, the main groups were carboxylic acids (>36%) and hydrocarbons (>24%), whereas ethers (≈32%) and hydrocarbons (≈29%) were the predominant substances in the SK group (Figure 1).

### 2.3. Sensory Parameters of Dry Fermented Sausages

The results of the sensory evaluation of fermented sausages are summarized in Table 3. The addition of chia seed in the amounts of 1% and 2% as well as 1% cumin seed presented similar overall quality obtained for control sausage. Significant lower overall quality was noted for sample with 2% addition of cumin seed. No significant differences among treatments were indicated in case of meat color, hardness, sour odor and flavor, and bitter flavor. Herbal odor and flavor was the highest in the samples with the addition of cumin seed. On the other hand, these samples showed significantly lower values of the rancid odor compared to the other sausages.

## 3. Discussion

### 3.1. Sensory Characteristic of Fermented Sausage with Particular Emphasis on Odor and Flavor

The process of the fermentation of sausages are aimed, apart from insuring product safety and extending the storage time, at giving specific flavor and odor characteristics. It is expected that the addition of chia and black cumin seed will increase the attractiveness of the product offered to consumers and will provide the health-promoting effect. The general sensory quality of the sausages with seed addition was high except for the sample with 2% addition of black cumin. The addition of 2% black cumin appears to be too high in terms of sensory characteristics. The meat color and hardness scores did not differ statistically significantly between the treatments. However, significant differences were observed regarding the odor and flavor of the sausages. As reported by Domínguez et al. [19], the main volatile compounds influencing the aroma of the final product belong to different chemical families, however not all these compounds have the same importance on the overall aroma perception. Alcohols have an important impact on the aroma of dry fermented meats due to their low odor threshold as they are involved in many metabolic pathways, including lipid oxidation, metabolism of amino acids, and reduction of methyl ketones [20]. The unpleasant flavor of rancid fat in food is a result of the release of low-molecular volatile compounds—i.e., short-chained aldehydes—or the resulting oxidation-induced acids [21]. The scores indicated for rancid odor were low but varied between treatments. According to the panelists, the least noticeable rancid odor was in the 2% black cumin sample, while the most noticeable was in the 2% chia sample. Rancid flavor was indicated by the panelists as the highest for the control sample and the sample with the addition of 1% chia seed. According to our previous study [10], incorporation of chia and black cumin seed did not affect lipid peroxidation of fermented sausages at the end of production and after 30 days of storage.

### 3.2. Volatile Compounds

Volatile compound composition of fermented sausages might be due to seasoning or the reactions between lipids, proteins, and carbohydrates caused by microbial or endogenous enzymes [12]. Alcohols are crucial in the aroma development of dry fermented meat products. SBC samples presented the lowest amount of total alcohols, whereas the SK samples achieved the highest. SK samples were rich in alcohols derived from the amino acid degradation: 3-methyl-1-butanol (fruity, whiskey notes), 2-methyl-1-butanol (fruity, winey), and phenylethyl alcohol (floral, rose, dried notes). On the contrary, similar amounts were observed in both types of seed treatments. The compound 2,3-butanediol (creamy, fruity, buttery), produced by the carbohydrate fermentation was significantly higher in SK batch, followed by SCh and SBC groups. Moreover, the formation of this compound was reduced by half when the percentage of chia seeds was higher. Regarding the lipid oxidation compounds, isopropyl alcohol (musty, alcohol), 2-pentanol (fermented, fruity, floral notes), and 1-hexanol (herbal, ethereal, fruity notes) were more abundant in SK samples. In contrast, 1-pentanol (sweet, balsamic, unpleasant aroma) derived from the degradation of lipid hydroperoxides was predominant in SCh 2%, whereas the lowest content was detected in SBC samples. These results are in agreement with the results of the sensory analysis as the samples with the addition of black cumin seeds were characterized by the lowest rancid flavor. The addition of a lower percentage of chia seeds decreased the levels of this compound, although a greater dose the effect is the opposite (48.61 vs. 131.11 AU × 10^3^ g^−1^ for 1% and 2% of chia seeds, respectively).

Aldehydes constitute one of the most important compounds that affect aroma due to their low odor threshold values. The lactic acid bacteria are capable of producing a large number of aldehydes such as 2-methylbutanal, 3-methylbutanal, pentanal, hexanal, and heptanal [17]. There were no significant differences in 2-methylpropanal (nutty, pungent) between treatments, so the breakdown of the amino acid valine was not influenced by the different types of sausages. Presumably, the addition of 2% of chia seeds could accelerate the amino acid degradation since the highest amounts of 3-methylbutanal, 2-methylbutanal, 3-ethylbenzeneacetaldehyde, benzaldehyde, and benzeneacetaldehyde were reached. However, the content of benzaldehyde (almond, burnt sugar) and benzeneacetaldehyde (honey-like) from the phenylalanine amino acid degradation improves the flavor of dry sausages [22]. In addition, 3-methylbutanal (roasted notes) is considered one of the strongest odorants in dry-fermented sausages [23]. The addition of 2% chia seeds could also promote the degradation of lipids because there was an increase in the total number of compounds compared with the rest of the batches. Moreover, aldehydes derived from the oxidation of linoleic acid—such as butanal, pentanal (pungent notes), and hexanal—achieved the highest values. These findings are consistent with the TBARs values reported in our previous study, where SCh 2% suffered more lipid degradation than the other batches (2.53 mg kg^−1^) [10]. Hexanal has been used as a marker of quality and oxidative stability in meat and meat products. It is derived from the secondary oxidation of ω-6 PUFA [24]. At low levels, it provides pleasant green notes, though at high amounts it gives an unpleasant rancid aroma [19]. Another product of ω-6 fatty acids oxidation is heptanal (oily fruity) [25]. The maximum levels of this compound were achieved in SK samples, followed by SCh 2%. SBC sausages were rich in propanal (pungent), a typical degradation product of the ω-3 fatty acids. Indeed, it was the dominant aldehyde in this type of formulation. Black cumin seeds and their essential oils are a good source of linoleic (ω-6) and oleic (ω-9) acids. They also contain linolenic acid (ω-3), although in a lower percentage. Some authors reported that linoleic acid was in a proportion above 53%, oleic acid varied from 22% to 28%, and linolenic acid was less than 1% [26,27,28]. The high molecular weight aldehydes like pentadecanal contribute to the flavor development because they act as precursors to alkanals and alkenals [29]. This compound was significantly lower in SK samples. Chia seeds have highly polyunsaturated lipids, where the primary fatty acid is α-linolenic acid, which represents more than 60% of PUFA. It also contains roughly 20% of linoleic acid [30]. Despite having a nutritionally favorable fat composition, a high PUFA concentration results in high instability, triggering lipid oxidation [31]. Moreover, chia seeds are a good source of natural antioxidants such as carotenoids, phytosterols, polyphenolic compounds, and tocopherols [32]. The higher percent of chia seeds could act as prooxidant, since a lower percent of chia entailed less formation of lipid oxidation compounds.

The origin of carboxylic acids is mainly due to the oxidation of unsaturated fatty acids and the hydrolysis of phospholipids ad triglycerides [33]. Only four compounds belonging to this family were found, where the SK samples presented the lowest amount. There were no statistically significant differences in butanedioic acid, phenyl- and propanoic acid anhydride. The incorporation of the seeds led to a greater formation of the short-chain organic acids (containing less than six carbon atoms). Owing to their low odor threshold, they impart powerful aromas to meat products [19]. Butanoic acid (unpleasant fermented, rotten cheese-like) and acetic acid (pungent, sour) are products derived from carbohydrate fermentation produced by LAB. As they give off very potent odors, they are intimately involved in the typical aroma of fermented sausages [23]. Sausages formulated with seeds have more levels of these acids. The presence of butanoic acid could also derived from valine deamination [34]. This compound was in similar amounts in all sausages formulated with seeds with values ranging from 686.99 to 773.81 AU ×10^3^ g^−1^. Considering the acetic acid data, similar values were found in SCh sausages (≈15,744.23 AU × 10^3^ g^−1^). A greater amount of black cumin seeds resulted in an increased of this substance, which has a very characteristic vinegar odor (10,8061 vs. 14,797.66 AU ×10^3^ g^−1^ for SBC 1% and SBC 2%, respectively). In our previous study, it was deduced that the addition of both chia and black cumin seeds promoted the growth of LAB [10].

A total of seven esters were identified in the sausage samples. Compounds belonging to this chemical group are highly fragrant substances easily detected due to their low odor thresholds. Most of them were ethyl ethers, essential for attaining the fermented sausage flavor by giving very intense fruity notes masking rancid odors [35]. Low molecular weight esters can be linked to the esterase activity of certain microorganisms such as lactic acid bacteria, *Micrococcaceae*, and *Staphylococcus* spp. [19,35]. As it can be observed, the total amount of esters did not show significant differences (*p* > 0.05) among the sausages with different formulation. In addition, similar amounts of ethyl ethanoate (sweet, banana, glue), ethyl butyrate (caramel, pineapple, strawberry, sweet, floral), and ethyl lactate (popcorn, buttery, tart, fruity, floral) were achieved in all sausages. Ethyl isobutyrate (sweet, fruity) and 3-methylbutylacetate (sweet, banana) compounds were at significantly higher rates (*p* < 0.001) in SK group than in SCh and SBC batches. Nevertheless, ethyl propanoate (sweet, apple, tropical fruity) was found in similar amounts in all sausages except for SCh 2% group. The addition of a higher percentage of chia seeds resulted in a significant increase in the formation of this compound (about 5-fold higher). SK samples showed the highest amount of total ether (13,263.06 AU ×10^3^ g^−1^), principally due to their high dimethyl ether content (12,980.42 AU ×10^3^ g^−1^). Similar amounts of this alkane ether were detected in the other batches. However, SK sausages together with SBC sausages presented the lowest levels of the remaining ethers, excluding the cis-4-methoxy thujane. This compound was in the highest quantities in SBC sausages, especially in those with greater content of seeds. It is one of the main components of the essential oil of some varieties of black cumin seeds, such as those grown in Ethiopia [36]. 1-butoxy-2-propanol, a lipid oxidation compound [37], did not show significant differences among samples (*p* > 0.05). Regarding the SCh sausages, the addition of more percentage of chia seeds entailed more accumulation of trimethylene oxide, 3,3-dimethyl-1,2-epoxybutane, 2-ethyl-furan, and 2-pentyl-furan. The two furanic compounds were presented in all sausages. These heterocyclic organic compounds are originated from the Maillard reaction, Strecker degradation, and thermal degradation of thiamine [38]. They are derived from the smoking process, imparting roasted nuances and pleasant aromas with fruity, green, sweet, and vegetable notes [39]. Due to their low odor thresholds, they are important odorants in dry-cured meat products [40]. Additionally, 2-ethyl-furan comes from the oxidation of ω-3 fatty acids, whereas the 2-pentyl-furan comes from ω-6 fatty acids [41]. The higher amount of these furans in SCh 2% could be explained by the oxidation of linolenic and linoleic acids, the main fatty acids present in chia seeds [42].

On the other hand, a significant reduction in the total amount of hydrocarbons was observed in the SBC 2% samples (7978.73 AU × 10^3^ g^−1^), whilst the other sausages showed similar values ranging from 11,373.17 to 13,201.3 AU × 10^3^ g^−1^ for SCh 2% and SCh 1%, respectively). It should be noted that branched hydrocarbons were the most abundant, with a total of 23 different compounds identified, followed by nine linear hydrocarbons, and two cyclic hydrocarbons. The addition of more percentage of seeds led to a diminution of the branched hydrocarbons. Moreover, the lowest number of lineal hydrocarbons was found in the SBC 2%. On the other hand, no differences were observed among samples in the total of cyclic hydrocarbons (*p* > 0.05). Despite a large number of the compounds detected belonging to this category, they are not of great interest due to their relative high threshold of odor perception [43].

In the present study, the total amount of ketones was significantly higher (*p* < 0.05) in the control samples mainly due to the addition of seeds led to a reduction of cyclobutanone compound. However, sausages formulated with chia seeds had the highest content of almost all remaining ketones. Indeed, a higher percentage of chia seeds produced a significant increase (*p* < 0.05) in the content of those ketones. Contrariwise, regarding the 2-heptanone, a degradation product of linoleic acid, was significantly most abundant in SBC sausages (*p* < 0.05). Both linear and methyl ketones are derived from the degradation of free fatty acids. Furthermore, methyl ketones contribute to the fatty aroma of meat products, which can be also produced by β-oxidation or β-keto acid decarboxylation of SFA [44,45]. The 2-ketones detected play an important role in the aroma due to their peculiar blue cheese aroma [35]. Acetoin, mainly produced by the microbial carbohydrate fermentation predominated by LAB [46], was significantly higher in SCh samples, concretely in the addition of 2%. Due to its very low odor threshold and characteristic buttery odor, it has a great importance on the product aroma. 2-Butanone is another by-product of LAB metabolism [47]. The higher content of this ketone in sausages with seeds is consistent since, as previously mentioned, the growth of LAB was higher in this batch than in control group. Another important ketone that arose from lipid autooxidation was the 3,5-octadien-2-one with characteristic mushroom and woody notes. This compound was significantly higher (*p* < 0.05) in the SCh 2% sausages, showing similar levels in the rest of the samples.

On the other hand, a total of 16 monoterpenes (one acyclic, eight monocyclic, and seven bicyclic) and 2 tricyclic sesquiterpenes were identified. Additionally, three of them are monocyclic aromatic hydrocarbons (o-cymene, m-cymenene, and thymol), which have an important impact on the overall aroma due to their lower threshold odor [33]. As it was expected, no terpenoids were detected in the control samples, since the presence of these compounds is due to the addition of these seeds. However, terpenes such as limonene or terpinene can appear in meat products due to their inclusion in the animal diet [48]. Particularly, SBC sausages had the greatest amount of all individual terpenes comparing with SCh group. Moreover, a greater addition of the black cumin seeds showed higher amounts of the terpenoids, excepting o-cymene. Seven monoterpenes (α-phellandrene, α-thujene, α-pinene, β-pinene, D-limonene, o-cymene, and γ-terpinene) of the 18 terpenes identified were detected in the SCh samples. In this case, the higher contribution of chia seeds led to an increase in the content of all individual terpenes. Furthermore, for the same percentage of seeds, black cumin provided the highest amount of the total terpenoids. Indeed, it was around 88 times higher for the formulation with 1% and roughly 59 times for the 2%. Some authors also found β-pinene, α-pinene, D-limonene, and p-cymene in the chia seed oil [49]. The major constituents of the black cumin sausages were the o-cymene and α-phellandrene, both representing around 55% of the total volatile compounds. These compounds could be a biomarker of this kind of black cumin, since the seeds employed are rich in these compounds. Moreover, *Nigella sativa* is a good source of other bioactive compounds such as terpinen-4-ol (minty), α-pinene (herbal, fresh, woody), longifolene (unpleasant), thymol (herbal, medicine), α-thujene (citrus, sweet, sour), and β-pinene (pine, pungent) [50,51]. Other terpenoids were also identified in back cumin including sabinene (woody, citrus), d-limonene (citrus), γ-terpinene (citrus), α-terpinolene (fruity, green, pine), α-longipinene (pine), β-myrcene (herbaceous, mint) [36,52]. All of the above compounds were detected in SBC sausages in appreciable amounts. On the other hand, γ-terpinene detected in the SBC sausages is the precursor of thymoquinone (TQ). It suffers dehydrogenation and aromatization, leading to the formation of p-cymene. Then, this compound is converted to thymol through hydroxylation, and finally, it is oxidized to TQ [53]. Different biological activities have been reported for most of the compounds identified in this study, such as antimicrobial, antioxidant, anti-inflammatory, and anticarcinogenic properties, among others [54].

A total of seven substances were enclosed in other compounds: one halogenated derivative, three sulfurous compounds, and three nitrogen compounds. The lower quantity of the total compounds was achieved in SK samples. The sulfur-derivatives carry a greater weight than the others since they represent 78% in SK group and more than 87% in the seed batches. They are compounds of special interest due to their low-odor threshold. Moreover, they have been detected in dry-cured meat products as important odorants [40]. The sulfur-containing volatiles are originated from the catabolism of amino acids during the fermentation process [55]. The amount of carbon disulfide (rotten-egg notes) in seed sausages was higher than the other sulfur compounds, especially in the formulation with black cumin (≈ 535 AU × 10^3^ g^−1^). By contrast, SBC samples had the lowest content of dimethyl sulfone (134 AU × 10^3^ g^−1^), while the rest of the batches presented similar amounts with values ranging from 219.78 to 248.35 AU × 10^3^ g^−1^ for SK and SCh1% batches, respectively. This compound is naturally widely present in green plants, fruits, vegetables, grains, animals, etc. [56,57]. It has been also associated with unfavorable sensory descriptors imparting burnt and sulfur notes [58]. On the other hand, methanethiol was predominant in SCh sausages reaching values around 17.48 AU × 10^3^ g^−1^. Regarding 2,3-dimethyl-arizidine, no significant differences were found among the batches (*p* > 0.05).

## 4. Materials and Methods

### 4.1. Dry Fermented Sausage Preparation

The dry fermented sausages produced with 50 mg kg^−1^ sodium nitrite addition were the material tested in the experiment. The experimental meat products were manufactured from ham muscles from Polish large white purebred fatteners obtained from a local slaughterhouse at 48 h postmortem. The meat was minced through a 0.01 m grinding plate using the grinder (KU2-3EK, Mesko-AGD Skarzysko-Kamienna, Poland). While mixing the ingredients, glucose (0.6%) and 2.8% of curing mixture (sea salt + sodium nitrite) were added to each formulation. The composition of the curing mixture and the amount of its application ensured the presence of sodium nitrite in the stuffing in the amount of 50 mg kg^−1^. The amount of sodium nitrite was reduced in relation to the permitted amount in accordance with the Commission Regulation (EU) No. 1129/2011. The amount of nitrite permitted for use in cured meat products is currently 150 mg kg^−1^.

Grounded *Salvia hispanica* (Chia) seed and *Nigella sativa* (Black Cumin) seed were also used in the experiment. Five different formulations of the sausages were prepared: SK—control sample; SCh 1%—sample with 1% addition of chia seed; SCh 2%—sample with 2% addition of chia seed; SBC 1%—sample with 1% addition of black cumin seed; SBC 2%—sample with 2% addition of black cumin seed. Sausages were manufactured in semi-technical conditions with the technology previously described [10]. The fermentation and drying process was carried out in fermentation chambers (ITALFROST-DE RIGO-GS, Pszczyna, Poland) for 30 days under controlled humidity and temperature conditions. The production process consisted of three stages and is characterized by the following conditions: stage 1: T 20–22 °C, RH 55–63%, 3 days; stage 2: T 14–16 °C, RH 68–75%, 3 days; stage 3: T 13 °C, RH 76%, 24 days.

### 4.2. Proximate Chemical Composition

Proximate chemical composition of dry fermented sausages (collagen, moisture, protein, and fat contents) were determined using a Food Scan Lab 78,810 (Foss Tecator Co., Ltd., Hillerod, Denmark). Approximately 200 g of a homogenized sample (each) was distributed in the instrument’s round sample dish and loaded into the instrument’s sample chamber.

### 4.3. Determination of Volatile Compounds

The determination of volatile compounds was performed according to the procedure described by Pérez-Santaescolástica et al. [59]. Previous to the analysis, all samples were minced separately to produce a homogeneous and representative mixture. The extraction was conducted using a solid-phase microextraction. One gram of each sample was introduced into a 20 mL screw cap vial. Samples were conditioned at 37 °C during 15 min ensuring a homogeneous temperature. Subsequently, the extractions were carried out introducing the SPME fiber into the vial for 30 min. Afterwards, the fiber was transferred to the injection port of the GC-MS equipment. The fiber core was StableFlex (10 mm length) coated with 50/30 µm thickness of DVB/CAR/PDMS (divinylbenzene/carboxen/polydimethylsiloxane). The chromatography system 7890B (Agilent Technologies Santa Clara, CA, USA) coupled to mass spectrometer 5977B (Agilent Technologies) was employed to separate the compounds. The capillary column used was a DB-624 (30 m length × 0.25 I.D. mm × 1.40 µm film thickness; J&W Scientific, Folsom, CA, USA). The carrier gas employed was helium with a constant flow of 1.2 mL min^−1^ (9.59 psi). The ramp of temperature was firstly isothermal for 10 min at 40 °C, then raised to 200 °C at a rate of 5 °C min^−1^, next to 250 °C at 20 °C min^−1^ and held for 5 min. The total run time for analysis was 49.5 min. Mass spectra were obtained employing the mass selective detector working in electronic impact at 70 eV, with and electron multiplier voltage of 1275 V and collecting data at 2.9 scans/s over the range m/z 40–550 in scan acquisition mode. Compounds were identified by comparing the mass spectra obtained with those collected in the standard reference database NIST14 (National Institute of Standards and Technology, Gaithersburg, MD, USA), considering over than 85% of match factor. Results were expressed as area units (AU × 10^3^ g^−1^ of sample). Volatile compounds were measured in triplicate.

### 4.4. Sensory Analysis

The sensory analysis of the dry fermented sausages was assessed according to the method described in ISO/DIS 13299.2:1998 [60]. The sausages were sliced to an approximately 2 mm thickness with an electric slicing machine and placed in plastic odorless transparent boxes covered with lids. Sausages were cut into slices (2 mm thickness) and were presented to scientific staff (nine members, aged 30–50 years) from the Department of Animals Food Technology at the University of Life Sciences in Lublin (Poland). Samples of each sausage were blinded, served in containers with a unique sample number. A linear (100-mm) line graphic scale which was converted into conventional units (c.u.) was used for the assessment of sensory properties. The parameters analyzed and the descriptors used for each of them were as follows: meat color—from gray to pink; juiciness—from dry to juicy; and hardness and overall quality—from low to very high. For odor (intensity of rancid odor, sour odor, herbal odor), and flavor (intensity of rancid flavor, sour flavor, metallic flavor, herbal flavor, bitter flavor) descriptors ranging from ‘none’ to ‘very high intensity’ were adopted. Constant temperature, lighting, and elimination of distracting factors, such as noise and off odors, were applied during analysis.

### 4.5. Statistical Analysis

The IBM SPSS Statistics 25.0 software package was used to perform the statistical analysis. Normal distribution and variance homogeneity were previously tested (Shapiro–Wilk). The effect of different treatments was analyzed through a one-way analysis of variance (ANOVA), where the treatments and volatile compounds were taken to be as fixed and independent variables, respectively. Duncan’s multiple range test was applied to compare means when a significant effect (*p* < 0.05) was detected.

## 5. Conclusions

The results found in the present study confirmed that the *Salvia hispanica* and *Nigella sativa* seed have a significant effect on fermented sausages, since they contribute to the amount of volatile compounds as well as sensory features. The sausages with chia seed in the amount of 1% and 2%, as well as sample with 1% addition of black cumin seed, were characterized by positive sensory features, and their overall quality was rated on above 7 c.u. on a 10-point scale, similar to control sausage. The main groups of volatile compounds identified in fermented sausages are terpenes. The incorporation of black cumin in the formulation of fermented sausages caused the release of a greater amount of volatile terpenes. These compounds have a high influence in the aroma of fermented sausages. The intensity of the herbal odor and herbal flavor was rated the highest for the sausage with the addition of 2% black cumin, which resulted in the lowest overall quality of this sausage sample. Therefore, the use of even 2% chia seed had a positive effect on the quality of fermented sausages, while the addition of 2% black cumin caused adverse sensory changes.

## Figures and Tables

**Table 1:** Proximate chemical composition of dry fermented sausages.

Component	SK	SCh 1%	SCh 2%	SBC 1%	SBC 2%
Fat	13.32 ± 1.15 a	16.48 ± 1.42 b	16.96 ± 1.46 b	16.14 ± 1.41 b	17.05 ± 1.36 b
Protein	51.01 ± 1.21 c	52.52 ± 1.25 c	52.44 ± 1.24 c	36.28 ± 0.86 b	29.93 ± 0.71 a
Moisture	29.84 ± 1.2 a	29.06 ± 1.18 a	30.23 ± 1.23 a	36.26 ± 1.46 b	39.78 ± 1.62 c
Collagen	1.95 ± 0.32 a	2.97 ± 0.81 a	2.65 ± 0.65 a	1.90 ± 0.83 a	1.85 ± 0.72 a
Salt	6.17 ± 0.54 a	6.38 ± 0.49 a	5.48 ± 0.71 a	6.09 ± 0.56 a	5.94 ± 0.67 a

SK—control sample; SCh 1%—sample with 1% addition of chia seed; SCh 2%—sample with 2% addition of chia seed; SBC 1%—sample with 1% addition of black cumin seed; SBC 2%—sample with 2% addition of black cumin seed. Means with different lowercase letters (a–c) differ significantly (*p* < 0.05).

**Table 2:** Content of volatile compounds (AU × 10^3^ g^−1^ of sample) in dry fermented sausages.

**Family**	**Compound**	***m*** **/*z***	**LRI**	**SK**	**SCh 1%**	**SCh 2%**	**SBC 1%**	**SBC 2%**	**SEM**	**Sig.**
Alcohols	Glycidol	44	481	137.62 b	96.15 a	188.33 c	80.22 a	106.47 ab	8.97	***
	Isopropyl alcohol	45	514	282.02 c	155.72 b	101.02 ab	63.28 a	40.28 a	18.33	***
	2-Methyl-1-propanol	43	636	401.65 b	15.18 a	10.19 a	2.74 a	1.58 a	32.63	***
	2-Pentanol	45	743	111.22 b	47.70 a	50.42 a	32.19 a	26.19 a	6.67	***
	3-Methyl-3-buten-1-ol	68	796	30.50 b	38.90 c	40.86 c	9.94 a	5.09 a	2.86	***
	3-Methyl-1-butanol	55	801	1464.56 b	125.61 a	105.12 a	20.38 a	8.24 a	106.64	***
	2-Methyl-1-butanol	56	805	499.76 b	28.56 a	22.70 a	4.38 a	1.52 a	37.37	***
	1-Pentanol	55	841	90.77 bc	48.61 ab	131.11 c	17.58 a	4.29 a	10.93	***
	2,3-Butanediol	45	906	2473.58 c	1204.35 b	621.19 ab	98.95 a	3.07 a	197.16	***
	1-Hexanol	56	951	174.55 d	82.89 b	128.20 c	27.68 a	18.86 a	11.67	***
	Benzyl alcohol	108	1128	177.03 c	162.15 bc	147.50 b	51.99 a	35.91 a	11.24	***
	Phenylethyl alcohol	91	1187	95.13 b	31.13 a	24.76 a	7.84 a	3.51 a	7.76	***
	Total alcohols			5938.40 c	2036.95 b	1571.40 b	417.17 a	255.00 a	389.49	***
*Aldehydes*	2-Methylpropanal	72	540	12.32	10.32	14.68	11.8	15.46	0.65	ns
	Butanal	72	569	3.54 a	3.18 a	9.55 b	1.21 a	0.95 a	0.67	***
	3-Methylbutanal	58	649	92.44 a	92.59 a	149.74 b	68.66 a	76.13 a	7.02	***
	2-Methylbutanal	58	663	42.01 a	49.26 ab	64.41 b	39.96 a	43.10 a	2.90	*
	Pentanal	57	720	179.70 a	362.99 a	1431.66 b	14.73 a	10.51 a	117.37	***
	Hexanal	56	861	1979.82 b	600.15 a	2266.79 b	366.30 a	150.31 a	217.12	***
	Heptanal	70	972	189.67 c	57.88 a	103.99 b	49.47 a	22.88 a	12.31	***
	Benzaldehyde	106	1045	60.27 a	114.67 b	153.03 c	73.53 a	60.25 a	6.98	***
	Propanal	58	1073	10.06 a	24.58 a	21.98 a	3040.14 c	1597.42 b	263.24	***
	Benzeneacetaldehyde	91	1122	71.40 ab	50.36 a	84.62 b	63.42 ab	63.74 ab	3.75	*
**Family**	**Compound**	***m*** **/*z***	**LRI**	**SK**	**SCh 1%**	**SCh 2%**	**SBC 1%**	**SBC 2%**	**SEM**	**Sig.**
	3-Ethylbenzeneacetaldehyde	133	1215	2.68 a	5.73 a	14.87 b	1.69 a	1.35 a	1.12	***
	Pentadecanal	82	1530	6.67 a	16.22 c	13.12 b	10.58 b	10.48 b	0.69	***
	Total aldehydes			2650.58 abc	1387.93 a	4328.44 c	3741.47 bc	2052.57 ab	316.86	*
Carboxylic acids	Acetic acid	60	679	1770.23 a	15,484.76 c	16,003.69 c	10,861.63 b	14,797.66 c	1025.12	***
	Butanoic acid	73	913	472.87 a	694.52 b	773.81 b	697.67 b	686.99 b	22.85	***
	Butanedioic acid, phenyl-	104	951	21.95	24.24	19.85	18.65	20.86	0.67	ns
	Propanoic acid anhydride	57	1065	308.55	335.01	312.02	327.68	307.77	14.31	ns
	Total carboxylic acids			2573.61 a	16,538.52 c	17,109.37 c	11,905.62 b	15,813.28 c	1042.31	***
Esters	Ethyl ethanoate	43	584	2050.51	1906.52	1357.93	1217.62	957.25	144.52	ns
	Ethyl propanoate	57	728	121.23 a	75.00 a	370.81 b	23.39 a	11.63 a	29.85	***
	Ethyl isobutyrate	116	794	14.77 b	3.01 a	2.12 a	1.31 a	0.23 a	1.24	***
	Ethyl butyrate/Ethyl butanoate	88	849	482.17	243.04	210.19	208.05	178.27	37.73	ns
	Ethyl lactate	45	889	787.15	1015.63	1135.63	512.55	485.76	89.,91	ns
	3-Methylbutylacetate	70	936	45.05 b	12.61 a	11.33 a	6.31 a	5.29 a	2.97	***
	Oxalic acid, butyl cyclobutyl ester	55	1184	10.4	9.33	8.78	10.08	10.58	0.41	ns
	Total esters			3511.28	3265.13	3096.81	1979.32	1649.01	260.68	ns
Ethers	Dimethyl ether	45	499	12,980.42 b	4753.12 a	4737.93 a	4162.37 a	2850.05 a	989.60	**
	Trimethylene oxide	58	508	42.61 a	46.89 a	244.81 b	13.44 a	25.18 a	19.58	***
	2-Ethyl-furan	81	694	10.55 a	40.94 a	152.60 b	5.82 a	3.85 a	12.54	***
	3,3-Dimethyl-1,2-epoxybutane	70	778	3.44 a	3.85 a	16.99 b	1.32 a	0.41 a	1.41	***
	1-Butoxy-2-propanol	87	1010	192.1	138.89	122.02	79.59	52.72	18.78	ns
	2-Pentyl-furan	81	1036	33.37 a	44.38 a	124.58 b	49.32 a	63.90 a	7.96	***
	cis-4-methoxy thujane	153	1154	0.58 a	6.52 a	9.92 a	1663.94 b	2874.28 c	219.91	***
	Total ethers			13,263.06 b	5034.60 a	5408.85 a	5975.80 a	5870.40 a	913.42	*
Hydrocarbons	Pentane	42	497	566.89 b	13.95 a	20.93 a	185.81 a	127.00 a	58.33	**
	Pentane, 3-methyl-	57	534	2.98 a	6.92 a	20.57 b	3.71 a	1.76 a	2.08	*
	n-Hexane	56	544	490.80 a	1028.76 ab	1888.38 b	959.12 ab	574.69 a	162.51	*
	Isobutane	42	637	212.93 b	10.11 a	7.10 a	1.90 a	0.59 a	16.94	***
	Heptane	57	663	101.80 a	98.09 a	175.62 b	57.53 a	60.85 a	10.05	***
	Octane	85	814	230.69 bc	273.00 c	420.07 d	121.95 ab	71.91 a	29.33	***
	Octane, 2,3-dimethyl-	98	900	21.82 bc	25.79 c	20.57 bc	15.93 b	8.95 a	1.35	***
	Heptane, 3-ethyl-	57	900	66.23 b	79.02 b	66.57 b	45.25 a	25.55 a	4.50	***
	Nonane	85	931	15.16	18.03	17.6	18.55 a	14.06 a	0.69	ns
	Butane, 2,2,3-trimethyl-	57	962	17.60 c	22.66 d	15.08 c	10.97 b	5.18 a	1.21	***
	3-Ethyl-3-methylheptane	85	984	21.21 cd	23.69 d	18.81 bc	17.64 b	8.01 a	1.08	***
	Octane, 3-ethyl-	71	1000	36.05 d	30.84 c	26.55 b	39.19 d	21.15 a	1.31	***
	Undecane, 6,6-dimethyl-	57	1002	24.96 a	26.89 a	24.21 a	34.28 b	23.03 a	0,93	***
	Nonane, 3-methylene-	70	1019	96.75 ab	111.92 b	83.36 a	169.33 d	132.73 c	6.03	***
	Decane	71	1028	155.99 a	998.98 b	781.27 ab	1187.57 b	255.46 a	120.53	*
	Hexane, 2,2-dimethyl-	56	1044	135.71 b	162.53 c	93.81 a	126.67 b	76.37 a	6.58	***
	2,2,4,4-Tetramethyloctane	57	1060	4058.55 b	4348.46 b	3115.53 a	4000.35 b	2752.89 a	162.57	**
	Dodecane, 2,6,10-trimethyl-	57	1067	1245.39 bc	1515.65 d	1020.92 ab	1277.64 c	863.18 a	53.27	***
	Tridecane, 6-methyl-	57	1072	240.31 c	242.09 c	200.19 b	34.12 a	25.42 a	18.69	***
	Undecane, 3,6-dimethyl-	57	1079	1132.69 c	1131.42 c	814.13 b	926.54 b	572.25 a	43.00	***
	Undecane, 3,5-dimethyl-	71	1079	444.29 b	451.81 b	332.49 a	411.86 b	297.98 a	13.04	***
	Hexane, 2,2,5-trimethyl-	56	1082	58.78 a	60.93 a	63.97 a	57.40 a	42.17 a	3.54	ns
	Pentane, 2,2,4-trimethyl-	57	1084	166.14 c	168.61 c	127.88 b	174.49 c	96.71 a	6.19	***
	Decane, 2,6,7-trimethyl-	71	1084	56.18 b	26.32 a	43.64 ab	40.23 ab	31.36 a	3.15	*
	Heptane, 3,3,4-trimethyl-	71	1088	178.24 b	203.41 b	178.85 b	182.57 b	113.31 a	7.00	***
	Heptane, 3-methyl-	56	1095	7.2	6.51	6.17	5.85	5.91	0.30	ns
	Cyclobutane, 1,1,2,3,3-pentamethyl-	70	1105	25.55	22.98	23.64	25.57	21.89	1.11	ns
	Undecane	57	1114	1171.85 b	1130.56 b	834.97 a	1140.39 b	828.49 a	35.20	***
	Decane, 3,3,5-trimethyl-	71	1128	71.4	73.52	69.75	93.29	86.6	3.39	ns
	Decane, 3,3,8-trimethyl-	57	1128	118.12	129.84	117.83	125.09	122.25	6.84	ns
	Octane, 2,3,3-trimethyl-	57	1158	41.99	36.98	31.47	42.39	32.86	2.37	ns
	Dodecane	57	1191	615.92	561.54	549.14	637.36	527.67	33.30	ns
	Cyclopentane, butyl-	83	1229	16.83 b	12.85 a	12.24 a	17.32 b	16.72 b	0.54	***
	Tridecane	57	1263	175.85 bc	147.08 ab	149.87 ab	192.83 c	133.80 a	6.19	**
	Total branched hydrocarbons			8455.52 b	8895.94 b	6499.45 a	7836.66 b	5346.20 a	300.73	***
	Total lineal hydrocarbons			3524.95 ab	4269.97 bc	4837.84 c	4501.11 bc	2593.93 a	222.12	**
	Total cyclic hydrocarbons			42.39	35.83	35.88	42.88	38.6	1.43	ns
	Total hydrocarbons			12,022.86 b	13,201.73 b	11,373.17 b	12,380.65 b	7978.73 a	413.91	***
Ketones	2-Butanone	72	579	15.13 a	24.42 bc	30.72 c	21.13 ab	20.23 ab	1.46	**
	2,3-Pentanedione	100	728	25.43 a	34.49 a	196.98 b	5.18 a	2.82 a	16.30	***
	Acetoin	45	782	85.71 a	187.05 ab	263.31 b	114.53 a	106.11 a	21.06	*
	Cyclobutanone	70	801	1039.22 b	92.72 a	69.96 a	13.77 a	5.41 a	76.55	***
	3,4-Hexanedione, 2,2,5-trimethyl-	57	893	4.20 b	4.71 b	4.65 b	3.87 ab	2.48 a	0.26	*
	2-Heptanone	58	965	26.34 a	16.95 a	33.12 a	56.07 b	57.79 b	4.23	**
	3,5-Octadien-2-one	95	1138	2.39 a	14.17 a	95.67 b	2.43 a	1.69 a	8.52	***
	Total ketones			1198.43 c	374.50 a	694.41 b	216.98 a	196.52 a	74.85	***
Terpenoids	α-Phellandrene	93	967	0.00 a	256.18 ab	440.91 b	43,144.03 c	46,901.02 d	4081.00	***
	α-Thujene	77	967	0.00 a	82.27 a	135.61 a	16,026.38 b	18,933.94 c	1593.57	***
	α-Pinene	93	975	0.00 a	340.86 b	591.76 c	7423.01 d	9565.12 e	756.40	***
	Dehydrosabinene	91	993	0.00 a	0.00 a	0.00 a	362.01 b	496.82 c	39.94	***
	Sabinene	93	1019	0.00 a	0.00 a		4685.18 b	6208.70 c	511.49	***
	(-)-β-Pinene	93	1021	0.00 a	127.34 a	207.24 a	6752.91 b	7041.00 b	623.97	***
	β-Myrcene	93	1030	0.00 a	0.00 a	0.00 a	134.53 b	257.13 c	19.25	***
	(+)-4-Carene	121	1058	0.00 a	0.00 a	0.00 a	2430.25 b	3712.91 c	289.94	***
	D-Limonene	68	1067	0.00 a	64.25 a	82.24 a	4701.64 b	7617.69 c	582.84	***
	o-Cymene	119	1071	0.00 a	988.55 a	1499.90 a	71,876.77 c	63,249.49 b	6160,39	***
	γ-Terpinene	91	1092	0.00 a	8.43 a	13.32 a	3339.94 b	5566.69 c	426.26	***
	α-Terpinolene	121	1117	0.00 a	0.00 a	0.00 a	337.77 b	558.84 c	43.08	***
	m-Cymenene	132	1131	0.00 a	0.00 a	0.00 a	1370.95 b	2068.40 c	162.17	***
	Thujone	110	1175	0.00 a	0.00 a	0.00 a	20.81 b	36.99 c	2.81	***
	Terpinen-4-ol	111	1213	0.00 a	0.00 a	0.00 a	160.03 b	286.70 c	21.81	***
	(E)-Longipinene	119	1324	0.00 a	0.00 a	0.00 a	236.66 b	455.97 c	34.11	***
	Thymol	135	1328	0.00 a	0.00 a	0.00 a	389.44 b	761.60 c	56.88	***
	Longifolene	161	1367	0.00 a	0.00 a	0.00 a	401.08 b	755.84 c	56.73	***
	Total terpenoids			0.00 a	1867.87 a	2970.97 a	163,793.39 b	174,474.86 c	15,279.80	***
Others	Methanethiol	72	579	11.52 ab	17.48 c	15.66 c	12.91 b	9.92 a	0.59	***
	Diazene, dimethyl-	100	728	33.30 ab	26.75 a	23.22 a	42.16 b	42.05 b	2.14	**
	Carbon disulfide	45	782	133.71 a	481.46 c	305.58 b	537.10 c	532.28 c	31.85	***
	Fumaronitrile	70	801	5.11 ab	11.46 b	22.35 c	2.36 a	1.71 a	1.73	***
	Butane, 1-chloro-3-methyl-	57	893	33.91 b	9.21 a	10.07 a	3.93 a	3.22 a	2.29	***
	Dimethyl sulfone	58	965	219.78 b	248.35 b	220.22 b	134.41 a	134.30 a	12.47	**
	2,3-Dimethyl-aziridine	95	1138	28.19 a	28.53 a	25.04 a	22.69 a	21.21 a	1.63	ns
	Total others			465.52 a	823.24 c	622.14 b	755.55 bc	744.70 bc	30.39	***
	Total compounds			41,623.72 a	44,530.47 a	47,175.56 a	201,165.95 b	209,035.07 b	14,687.11	**

SK—control sample; SCh 1%—sample with 1% addition of chia seed; SCh 2%—sample with 2% addition of chia seed; SBC 1%—sample with 1% addition of black cumin seed; SBC 2%—sample with 2% addition of black cumin seed. Means with different lowercase letters (a–e) differ significantly (*p* < 0.05). * *p* < 0.05, ** *p* < 0.01, *** *p* < 0.001, ns: not significant.

**Table 3:** Sensory parameters of dry fermented sausages.

Parameter (c.u.)	SK	SCh 1%	SCh 2%	SBC 1%	SBC 2%
Meat color	8.54 ± 1.48 a	8.26 ± 1.57 a	7.88 ± 1.64 a	7.58 ± 1.29 a	7.18 ± 1.66 a
Hardness	7.94 ± 1.33 a	7.78 ± 1.63 a	8.16 ± 1.09 a	8.44 ± 1.03 a	8.34 ± 1.18 a
Overall quality	8.16 ± 1.23 b	8.06 ± 1.40 b	7.28 ± 1.59 b	7.12 ± 0.52 b	5.28 ± 0.84 a
Rancid odor	0.80 ± 0.22 b	0.72 ± 0.14 b	1.16 ± 0.54 c	0.52 ± 0.15 b	0.24 ± 0.11 a
Sour odor	2.46 ± 0.56 a	2.68 ± 0.71 a	2.96 ± 0.63 a	1.76 ± 0.72 a	2.86 ± 0.32 a
Herbal odor	0.10 ± 0.02 a	0.44 ± 0.21 b	0.36 ± 0.11 b	5.42 ± 0.85 c	6.96 ± 0.38 c
Rancid flavor	0.60 ± 0.11 c	0.80 ± 0.20 c	0.10 ± 0.12 a	0.15 ± 0.09 a	0.40 ± 1.30 b
Sour flavor	0.15 ± 0.09 a	0.23 ± 0.11 a	0.18 ± 0.10 a	0.09 ± 0.10 a	0.18 ± 0.08 a
Metallic flavor	0.10 ± 0.07 a	0.12 ± 0.08 a	0.42 ± 0.14 b	0.10 ± 0.11 a	0.25 ± 0.10 ab
Herbal flavor	0.05 ± 0.07 a	0.05 ± 0.04 a	0.10 ± 0.07 a	3.40 ± 0.12 b	5.30 ± 0.18 c
Bitter flavor	0.01 ± 0.00 a	0.02 ± 0.01 a	0.01 ± 0.01 a	0.02 ± 0.01 a	0.01 ± 0.01 a

SK—control sample; SCh 1%—sample with 1% addition of chia seed; SCh 2%—sample with 2% addition of chia seed; SBC 1%—sample with 1% addition of black cumin seed; SBC 2%—sample with 2% addition of black cumin seed. Means with different lowercase letters (a–c) differ significantly (*p* < 0.05).

## Data Availability

Not applicable.

## Sample Availability

Samples of the compounds are not available from the authors.
